# Estimating non-communicable disease treatment costs using probability-based cost estimation

**DOI:** 10.1080/16549716.2021.2008627

**Published:** 2022-02-11

**Authors:** Claire R. Botha, Sten H. Vermund

**Affiliations:** a Health Systems Strengthening, The Aurum Institute, South Africa; b Yale School of Public Health, United States

**Keywords:** Probability-based cost estimation, non-communicable diseases, low-and-middle income countries, healthcare cost, South Africa

## Abstract

The burden and impact of non-communicable diseases (NCDs) are well documented, accounting for 70% of premature deaths globally. In Sub-Saharan Africa, rising NCDs are estimated to account for 27% of mortality by 2020, a 4% increase from 2005. This increase will inevitably lead to a higher demand for NCD treatment services, exerting pressure on limited public financial resources. To get a sense of the resources required to treat NCDs, it is necessary to estimate the costs associated with the diagnosis, treatment and management thereof. Typically, in estimating costs for health services, countries use historical patient level data combined with demographic trend data and non-patient level data to arrive at estimated future costs. This methodology relies heavily on the availability of data from a wide variety of sources stretching beyond the health sector. Low-and-middle-income countries often lack the requisite data and are compelled to use less efficient ways to determine resource allocation. This study explores the use of probability-based cost estimation to estimate the cost of delivering NCD treatment services in South Africa, one such data-poor environment.

Probability-based cost estimation, in combination with deterministic cost estimation, is used in arriving at a cost estimate for NCD treatment services at primary healthcare facility level. On its own, deterministic cost estimation can determine total costs, provided all the input variables are known. This is not always possible because of the lack of one or more input variables. In most instances, the lacking input variable is the quantities at which specific conditions will be treated. This problem is addressed by using probability-based cost estimation through which a mean cost is calculated and applied to the target population as a whole, eliminating the need for quantities per condition. Thus, this model contains both deterministic and probabilistic cost estimation elements.

## Background

Non-Communicable Diseases (NCDs) are the leading cause of premature death globally and are a growing, significant public health challenge in low- and middle-income countries (LMICs). In sub-Saharan Africa (SSA), NCDs account for a preponderance of deaths, due, in part, to a failure to prevent and/or mitigate their associated risk factors [1–4]. Among premature deaths, 80% are due to cardiovascular disease, cancer, respiratory disease and diabetes; among the world’s largest increases in these deaths are occurring in Africa [5–9].

The financial burden resulting from rapidly rising NCDs on the individual and household levels, and the health care delivery system in LMICs is well-documented [10–13]. With projected increases in the number of people living with chronic diseases, an increased demand for and utilisation of NCD-related health care services is a certainty. To understand these resource requirements, it is incumbent for costs to be estimated that are associated with the diagnosis, treatment and management of NCDs in resource-constrained LMICs [14,15].

Cost estimation is a data intensive exercise [16,17]. The type and quality of the data available have a direct impact on the accuracy of the estimate. In LMICs, critical data are not readily available for several reasons [18]:

- data are not being collected at all;
- data are collected but they are insufficient, and
- sufficient data are collected but are not readily accessible and/or require technical skills to process the large volume of data to make them accessible.

Probability-based cost estimation can mitigate some of these data challenges, specifically where the available data are insufficient and traditional deterministic methods of cost estimation cannot be employed. In this case study, we use probability-based cost estimation to arrive at cost estimates, given the absence of headcount and coverage data per condition. Headcount refers to the number of patients who visited primary health care (PHC) facilities, including new and follow-up visits for a particular period [19]. Coverage is the proportion of the number of persons receiving services in relation to the targets for the given time period. Thus, we illustrate with this case study, the use of probability-based cost estimation as a costing model to estimate the cost of rendering NCD treatment services at PHC facility level in the Republic of South Africa.

Probabilistic Methods are widely used in different settings in the health sector. While it is used to estimate costs, it can also be used in cost effectiveness analysis to determine the cost effectiveness in the treatment of specific conditions [20]. It is also used in health technology assessment where a probabilistic sensitivity analysis is conducted to facilitate decisions around the use of specific technologies [21].

## Methods

Considering that the objective is to estimate the cost of rendering NCD treatment services, the case study approach is best suited to illustrate our real-life context [22]. We estimated costs for a defined geography (South Africa), for a specific category of diseases (NCDs) and for the 2020 financial year.

The methodology used in this case study consisted of two distinct steps. Each step involved a particular technique designed to address a particular problem. The final cost estimates were arrived at by combining the two techniques. When compared to classical cost estimating techniques, this represented a hybrid approach combining two separate techniques.

## Step 1: deterministic cost calculation

The purpose of deterministic cost calculation is to estimate the cost associated with treating each NCD condition separately. This cost is referred to as the condition unit cost. The treatment of a condition typically consists of several visits. Each visit incurs a cost based on the resources consumed. The total cost of the treatment of a condition is therefore determined by aggregating the costs of the visits. It is deterministic because the cost of each visit is known or can be reasonably estimated [23–25].

The conditions used in this case study are taken from the *Standard Treatment Guidelines and the Essential Medicines List: Primary Health Care Level* (2018 edition). This document includes 58 adult and 40 child NCD conditions [26]. The PHC costs for each condition are estimated based on the number and types of visits the condition typically comprises and the components utilised during the visit. A condition can have one or multiple visits when a patient presents for a health consultation. The *SAHealthBenefits Repository* lists the prescribed number of visits per condition (https://www.sahealthbenefits.org.za/home) [27]. The repository also contains the components used for a typical visit. These components are the resource inputs for the model that are required for each visit.

The cost components of a visit consist of human resources, equipment, diagnostics, medicines and medical consumables. The data required for the costing were obtained from sources in the government sector. Cost estimates were calculated from a public sector perspective, with the focus on costs to be incurred by the government as the service provider responsible for implementation. The calculation used to determine the cost for a particular component depends on the component type. This means that the calculation used for human resource costs differs from that used in calculating equipment costs. While both human resources and equipment are used in the treatment of multiple conditions, the challenge is to determine the costs attributable to a specific condition. With human resources, this is done by considering the annual salaries of the staff involved and the total time spent on the treatment of the condition. Equipment costs, on the other hand, take into account the acquisition costs, the lifespan of the equipment and the duration of the treatment for which it is used. Medical consumables, diagnostics and medicines are consumed per treatment of a condition and are therefore applied in full. Details of the calculations used for each component are presented in the appendix.

The total cost for a visit is determined by adding the component costs calculated for that visit. Total condition unit cost is arrived at by adding up the total cost for each visit prescribed in the treatment of the condition. The appendix lists the detail of the calculations involved with determining this figure.

## Step 2: probability-based cost estimation

Under circumstances where needed data are available, given the total condition unit costs calculated above, the next step would simply be to estimate the total condition cost for a particular demographic by multiplying the total condition unit costs by the number of services to be rendered. The total condition costs equal unit costs times quantity (headcount). In this context, quantity is a function of the number of services rendered in the previous period and services targeted for the next period. Calculating the total condition costs as described is not always practical because of the unavailability of historical data and uncertainty about future service demand for a particular condition.

We addressed this uncertainty by using probability-based cost estimation. In probability-based costing, the specific condition takes less precedence whilst costs themselves become the key focus. The costs involved in the delivery of services range over several values; we considered the cost as a random variable over this range.

The purpose of probability-based costing (also referred to as probabilistic cost estimation) is to determine the probability that a particular cost in this range of possible costs will be incurred and to assess associated risks. There are two ways in which probability-based costing can be applied. Firstly, through the Monte Carlo Simulation that is by far the best known technique. Secondly, through Symmetric Approximation (SA) which is also sometimes referred to as ‘The Poor Man’s Monte Carlo Simulation’ [28]. The latter produces results which come close to that of the former, but without the cost of having to purchase expensive computer software. It is for this reason that SA is used in this case study.

### Processes for a symmetric approximation

#### Determine probability density function

A Probability Density Function (PDF) is determined for each of the components that makes up the cost of treating a condition [29–31]. Whilst computer software randomly assigns values to each PDF when using the Monte Carlo Simulation, the mean and variance associated with each PDF is manually calculated with SA. This is done by calculating high, low and most likely values for each component and choosing a shape for the PDF.

##### Calculate high, low and most likely values

The high and low values for each cost component were determined by ranking the total for each cost component per condition from the highest to the lowest. This was done for both the adult and child populations, as shown (Table 1). Conditions were grouped into the following five World Health Organisation (WHO) NCD categories:

- cardiovascular diseases (like hypertension);
- diabetes;
- chronic respiratory diseases (such as chronic obstructed pulmonary disease and asthma);
- cancer; and
- other conditions.

**Table 1.** Symmetric approximation

Cost Component	Adult					Child				
	Low	Most Likely	High	Variance	Mean	Low	Most Likely	High	Variance	Mean
**Human Resources**	9.04	1768.925	4232.6	750,149	2004	12.97	303.81975	2334.6	266,629	884
**Equipment**	0.07	3.23	7.59	2	4	0.08	0.326875	4.97	1	2
**Diagnostics**	0	94.3025	677.52	22,446	275	0	0	63.74	226	21
**Medical Consumables**	1.03	214.2875	1436.34	99,972	551	0	48.61625	474.6	11,363	174
**Medicine**	0	239.48875	862.4	33,031	367	0	32.535625	162.19	1227	65
**Total**				**905,600**	**3182**				**279,446**	**1146**
**Std Dev**					951.630					528.626
**Coefficient of Variation**					29.90%					46.12%

Using convenience sampling, five health professionals operating at PHC facility level were asked to rank the categories. The health care professionals were instructed to rank categories with the most frequently treated conditions higher than those containing conditions less frequently treated. The cardiovascular condition category ranked the highest for adults, while the ‘other conditions’ ranked highest for children. The mean value of each cost component for the cardiovascular category was then chosen as the most likely value in the case of the adult population group. The same was done using values from the ‘other condition’ category for the child population.

##### Choosing a shape for the PDF

The range of values for a particular cost element can assume one of four possible shapes. Each shape typically represents the uncertainty of the cost for the component:

- Uniform Distribution (all outcomes between high and low values are equally likely);
- Triangular Distribution (data are asymmetrical with a skewness value that is positive or negative);
- Normal Distribution (the data are symmetrically distributed on either side of the mean); and
- Beta Distribution (a versatile way to represent outcomes as percentages or proportions).

Triangular Distributions are the most appropriate shape under conditions where there is a lack of knowledge about the actual distribution of costs but where high, low and most likely values are available. For this reason, the Triangular Distribution was used as the shape for each of the cost components.

#### Calculate the mean and variance for each cost component

The average cost and the standard deviation for each cost component, using the Triangular Distribution, is calculated using the following formula.

$$Average\;Cost(mean)\;=\;(low+mostlikely+high)/3\sigma^{2}$$

$$Variance=\;\sigma^{2}$$

**Table:** 

µ Average Cost (mean) = (low + most likely + high)/3	σ^2^ Variance = σ^2^

Applying this formula, we arrived at a mean and variance for each component as shown in Table 1.

#### Estimate the mean and standard deviation for the total costs

The means and variances of each component were summed to arrive at the mean and standard deviation for the total costs. With adults, the mean and standard deviation amount to 3 182 and 951,630, respectively. For children, the mean and standard deviation is 1 146 and 528, 626, respectively.

Given this mean and standard deviation, the question is now what would be the distribution of the total costs. Like the Monte Carlo simulation, SA uses a normal distribution as a standard assumption. Also, any single NCD health condition typically has many independent cost items associated with it. As a result, we assume that *N* is large enough for our total cost distribution to be normally distributed.

The normal distribution can determine the unit cost at different probabilities. This, together with the total number of services, is used to determine the total costs.

### Determine the probability of budget over/underrun

As pointed out above, the unit cost can be determined as a probability of the normal distribution or it can be arrived at by some other method, e.g. in-house methodology using historical figures. Once a unit cost has been established, it can be used as a point of estimate (POE) for which possible budget over/underruns can be determined.

The POE will either fall to the left or right of the mean. Any value to the right of the POE indicates a budget overrun and values to the left an underrun. For example, if we take an estimate of 3 824 we can calculate budget over and underruns as follows:

- Determine the Z-value using the formula, Z = (POE- µ)/ σ = (3 824–3 182)/951.630 = 0.674632
- Using the Standard Normal Distribution Table, we find the probability associated with this value equals 0.2486 or 25%.

Because the POE falls to the right of the mean, the probability of a budget overrun is calculated as 50% minus the probability of the Z-value, in this case 50% minus 25% equals 25%. The probability of a budget underrun equals 100% minus the probability of an overrun, in this instance it is 75%. If the POE falls below the mean, then the probability of an overrun is determined by adding 50% to the probability of the Z-value.

### Estimating Services

Completing the steps outlined above resulted in a unit cost for the targeted probability. If the targeted probability is 50% then the unit cost equals ZAR 3 182 for adults. Estimating the total costs involves multiplying the number of projected services with the unit costs. The number of projected services is estimated using the following formula:

$$s=\left(P\left(1+i\right)\times u\times \left(\frac{1}{c+ci}\right)\right)/v$$

*Where: s is the number of services, v is the average number of visits for the most likely condition category; p is the population (adult or child) for a particular geography; i is population growth rate; u is utilisation rate; c is coverage; ci is coverage increase rate; coverage = (headcount /headcount target); coverage increase rate is the rate at which the increase in coverage is planned.*

We first adjusted population (p) by the projected growth in population (i) and then further adjusted with the utilisation rate and the planned increase in the utilisation rate. The result is the projected headcount, whose healthcare service visits are estimated by dividing it by the average number of visits (Table 2 and 3). Population numbers and the projected increase were obtained from Statistics South Africa (STATSSA) [32,33]. Utilisation rates and headcounts (clinic visits) were obtained from the NDoH.

**Table 2.** NCD treatment services for adults. Table 2 follows the projected services formula below

Province	Population	PHC Headcount	Uitilisation Rates	Services
Eastern Cape	4,615,040	11,841,466	2	1,337,043
Free State	2,078,879	4,073,217	2	602,281
Gauteng	11,797,965	15,689,380	2	2,563,529
KwaZulu-Natal	7,975,441	20,261,387	2	2,310,599
Limpopo	4,137,210	9,777,467	2	1,198,609
Mpumalanga	3,290,020	6,468,022	2	953,166
Northern Cape	918,972	2,020,281	2	266,239
North West	2,915,078	5,644,648	2	844,540
Western Cape	5,221,073	10,689,597	2	1,512,619
**Total**	**42,949,678**	**86,465,465**	**2**	**11,588,626**

STATSSA and DHIS; Population >20 yrs older and PHC Headcount > 20 older.

## Results

Using the mean and standard deviation from Table 1, the total costs of providing NCD treatment services are estimated in Table 5. The NORMDIST function in Excel can estimate the unit costs at various probabilities using the inputs, as shown in Table 4.

**Table 3.** NCD treatment services for children

Province	Population	PHC Headcount	Uitilisation Rates	Services
Eastern Cape	2,097,236	2,057,253	1	1,063,299
Free State	808,586	467,294	1	409,953
Gauteng	3,378,151	1,958,824	1	1,712,723
KwaZulu-Natal	3,313,645	3,505,659	1	1,680,018
Limpopo	1,845,374	1,630,380	1	935,605
Mpumalanga	1,302,167	1,046,369	1	660,199
Northern Cape	344,903	259,492	1	174,866
North West	1,112,082	729,783	1	563,826
Western Cape	1,623,199	1,588,620	1	822,962
**Total**	**15,825,343**	**13,243,674**	**1**	**8,023,449**

STATSSA and DHIS; Population 5–19; PHC Headcount 5–9 and 10–19.

**Table 4.** Normal distribution inputs

	Adult	Child
**Mean**	ZAR 3 182	ZAR 1 146
**Variance**	905,600	279,446
**Std. Dev**	951,630	528,626

**Table 5.** Total costs at different probabilities in 2020, in thousands of South African Rand and US Dollars

Probability	Population	Unit Cost (ZAR)	Over/Underrun (%)	Unit Cost (USD)	Services	Total Cost (ZAR)	Total Cost (USD)
**50%**	Adult	3182	50/50	177	11,588,626	36,87,50,07,932	2,05,31,57,128
	Child	1146	50/50	64	8,023,449	9,19,48,72,554	51,19,59,703
**75%**	Adult	3824	25/75	213	11,588,626	44,31,49,05,824	2,46,74,01,903
	Child	1503	25/75	84	8,023,449	12,05,92,43,847	67,14,44,532
**90%**	Adult	4402	10/90	245	11,588,626	51,01,31,31,652	2,84,03,51,249
	Child	1824	10/90	102	8,023,449	14,63,47,70,976	81,48,46,858

The final cost estimate is arrived at by multiplying the unit cost at a targeted probability by the total number of services.

At 50%, the probability of a budget overrun is exactly the same as that for a budget underrun. At 75%, where the unit costs equals ZAR 3 824 (USD 213), the probability of a budget overrun is 25%.

In total, ZAR 19581032000 (USD 1 299,413,864) has been budgeted for clinic health services by the National Treasury for the 2020 financial year (https://vulekamali.gov.za/2020-21/departments?phrase=&province=all&sphere=all). This amount represents the totals for each province and it covers the cost to treat NCDs. This amount is much lower than the cost estimates listed in Table 5, even when compared with the 50% probability funding level calculated at ZAR 46069880486 (USD 3 056 661 187). It suggests serious implications for service delivery, as pointed out in the discussion section.

## Discussion

This South African case study demonstrates that probability-based cost estimation can be a useful technique for estimating treatment costs when comprehensive data are lacking. We used probability-based cost estimation since condition level headcount and target data were not available. Although probability-based cost estimation is mainly associated with project management, its applicability in a health services research context becomes clear when a treatment plan is viewed in the same light as a project plan [34–36]. Like a project plan, a treatment plan has specific start and end points with several scheduled tasks and activities in between. This allows for the treatment of any condition to be viewed as a project with an associated cost. Furthermore, we are able to arrive at a total cost as an approximate normal distribution with a specific mean. This normal distribution represents the cost for a ‘generic condition’ which can then calculate the total treatment cost for all conditions.

The biggest strength of this technique is that it allows for cost estimation where data per condition is lacking, including the headcount, targets and prevalence. It does, however, rely on the availability of where data per condition are lacking, such as price lists for the cost components. It also requires the existence of standards for the duration of the treatment and the equipment, medical consumables and medicines used. These sources were available in South Africa but are often not available in LMICs. Prices of goods and services often differ between locations within a geography due to procurement, transportation, and handling costs. Although this technique does not cater for geographic price variations, it can be easily adjusted, with varying prices based on regional consumer price indexes (CPI) where available. The technique can also be used in multi-year costing by adjusting population numbers with the annual population growth rate and by adjusting prices with the annual CPI.

Another advantage of this technique is that it can be condition agnostic. Instead of trying to estimate the cost per condition, it sets out to calculate the cost at a specific PDF. The focus is therefore on the cost and not the condition. By decoupling the cost from the condition, the technique caters for deviations from the treatment standards, which are likely to occur in real life situations. For example, patients are seldom serviced for a single condition during a visit. Also, upwards or downwards changes in costs because of these deviations are likely to be covered depending on the PDF of the estimate. Finally, any imperfection in the data, such as misdiagnosis, could have a knock-on effect on the allocation of cost and therefore on the future projected cost estimates.

Limitations in our work are extant. Although this case study estimates the costs for two demographics, i.e. adult and child, other studies may well include more demographics defined by age groups at a more granular level. This will depend on the availability of disaggregated headcount data, and the availability of treatment standards per age group. The cost components used in this cost estimation were limited to costs directly associated with treatment. We therefore excluded other indirect costs, such as administrative salaries, maintenance costs and electricity that are incurred when rendering the health service. This can be considered as an overhead cost, which can be calculated as a percentage of the overall cost. The best way to do this is a subject for further investigation. We did not estimate the broader economic costs normally associated with the cost of illness analysis. Such an analysis would typically present the magnitude of the problem in terms of a cost estimate. With this case study, we sought to estimates the cost of resources required to deliver health services, specifically treatment costs. Case studies are vulnerable to having limited scientific generalisation because one cannot generalise from a single case. We nonetheless believe that this technique can find useful application in other scenarios with similar characteristics.

The technique presented in this study will resonate with policymakers, financial planners and health services providers, particularly those in the public sector, who are required to provide health services within the context of budgetary constraints and competing priorities. These cost estimates can feed into the National Treasury’s Medium Term Expenditure Framework and budgeting process in terms of financial resources required and directing resources towards NCD strategic priorities. For example, if National Treasury had used this technique, it would have been aware of the glaring underfunding for 2020, as illustrated in the results section. The impact of the underfunding for this period could mean that services will be rendered at a much lower standard as that prescribed by the Standard Treatment Guideline. It could also mean that far fewer services than anticipated will be rendered. Another possibility is that some diseases, especially those that require a lot of resources, may not get treated at all.

The technique is specifically useful in balancing budget underruns and overruns [37]. If a planner estimates the costs at a high probability function, it will increase the likelihood of a budget underrun. This may be accompanied by the risk of a budget overrun in other priority areas. The decision on where to set the probability is therefore taken in a context that takes different risks into account. Finally, probability-based cost estimation as a technique presented in this study contributes to the body of knowledge that uses statistical approaches in costing health services.

## Conclusions

Projecting future health costs relies heavily on the existence of adequate population, programmatic and cost data. The more accurate the data, the more credible the projected costs. Hence, the absence of adequate data has an adverse impact on projected future costs. To work around these challenges, probability-based cost estimation provides a viable alternative for estimating future costs in LMIC settings.

## Paper context

Estimating the costs of future health services relies heavily on the existence of historical data. Yet critical historical data are not always available or complete, especially in low- and middle-income countries. We demonstrate an application of probability-based cost estimation in combination with deterministic costing for non-communicable disease treatment in primary health care in South Africa.

## Appendix: Cost Component Calculations

Cost calculation methods that were used to calculate the cost of each component are presented below.

**Human Resources**: Costs are calculated by considering the type of human resources used and the duration of each visit. Only direct labour, i.e. those staff who are directly involved in the delivery of the service, is considered. The following formula is used to calculate human resource cost.

$$hc\;=\;\left(hcm\;x\;t\right)\;+\;\left(hcs\;x\;t\right)+\;\left(hcg\;x\;t\right)$$

*Where: hc is human resource cost; hcm is human resource cost per minute for a medical officer; hcs is human resource cost per minute for a professional nurse (speciality); hcg is human resource cost per minute for a professional nurse (general); t is time in minutes per visit.*

Human resource cost per minute for each type of human resource in this case study was sourced from estimates in the *SAHealthBenefits Online Repository* [38,39].

**Equipment**: Equipment is used multiple times across conditions and in several visits. The challenge is how much of the acquisition cost should be attributed to a particular visit and thereafter to the condition cost overall. In this case study, the straight-line depreciation method is applied to calculate the apportioned cost. This entails dividing the duration of the visit (minutes) with the lifespan in minutes multiplied by the acquisition price of the equipment, as represented in the following formula.

$$ec\;=\;\left(\sum hrm/LFP\;x\;525600\right)\;x\;aqc$$

*Where: ec is equipment cost; hrm is human resources in minutes; 525,600 is the number of minutes in a calendar year; LFP is lifespan; aqc is the acquisition price.*

The equipment for each visit was obtained from the *National Health Commodities Catalogue for Primary Health Care Facilities* (2018) and their corresponding prices come from the National Treasury: Office of the Procurement Officer, *Transversal Contracts* [40,41].

**Diagnostics**: The cost of diagnostic tests utilised during each visit is applied in full. The *National Health Laboratory State Price List* 2019/20 served as the source for the diagnostic test prices [42].

dgt = ∑i

*Where: dgt is total diagnostic test cost; i is cost for each diagnostic item.*

**Medical Consumables**: The cost of medical consumables utilised during each visit is applied in full. Data on medical consumables were sourced from the *SAHealthBenefits online repository*.

mc = ∑i

*Where: mc is total medical consumable cost; i is cost for each medical consumable item.*

**Medicine**: The cost of medicine utilised during each visit is also applied in full. Medication and the prices were sourced from the *National Department of Health, Primary Health Care Standard Treatment Guidelines and the Essential Medicines List* (6^th^ ed.) and the *Master Procurement Catalogue* (Green Book) [43].

mdc = ∑i

*Where: mdc is total medicine cost; i is cost for each medicine item.*

The visit costs are arrived at by applying the formula below (see above for definitions of terms).

vc = hc+ec+dgt+mc+mdc

**Total Condition Unit Costs**: The total condition unit cost can be represented by the following formula.

tcuc = ∑ vc

*Where: tcuc is the total condition unit costs; vc is the total cost for each visit.*
